# Transcriptome Profiling Reveals Genes Related to Sex Determination and Differentiation in Sugarcane Borer (*Chilo sacchariphagus* Bojer)

**DOI:** 10.3390/insects13060500

**Published:** 2022-05-26

**Authors:** Ao-Mei Li, Wei-Zhong He, Ji-Li Wei, Zhong-Liang Chen, Fen Liao, Cui-Xian Qin, You-Qiang Pan, Xian-Kun Shang, Prakash Lakshmanan, Miao Wang, Hong-Wei Tan, Dong-Liang Huang

**Affiliations:** 1 Key Laboratory of Sugarcane Biotechnology and Genetic Improvement (Guangxi), Ministry of Agriculture and Rural Affairs, Nanning 530007, China; liaomei1995@sina.com (A.-M.L.); csn899@aliyun.com (W.-Z.H.); wjl2004-7919@163.com (J.-L.W.); czl_good2007@163.com (Z.-L.C.); liaofen@gxaas.net (F.L.); qincuixian@126.com (C.-X.Q.); panyouqiang@aliyun.com (Y.-Q.P.); shanglei8289@163.com (X.-K.S.); plakshmanan2018@outlook.com (P.L.); gxwm2007@126.com (M.W.); 2 Guangxi Key Laboratory of Sugarcane Genetic Improvement, Nanning 530007, China; 3 Sugarcane Research Institute, Guangxi Academy of Agricultural Sciences, Nanning 530007, China; 4 Interdisciplinary Research Center for Agriculture Green Development in Yangtze River Basin, College of Resources and Environment, Southwest University, Chongqing 400716, China; 5 Queensland Alliance for Agriculture and Food Innovation, University of Queensland, St. Lucia, QLD 4067, Australia

**Keywords:** sugarcane borer, sex determination and differentiation, *Chilo sacchariphagus* Bojer, transcriptome

## Abstract

**Simple Summary:**

*Chilo sacchariphagus* Bojer is an important sugarcane pest globally. The identification of key genes associated with sex determination and differentiation will provide important basic information for the sterile insect technique control strategy. In this study, the comparative transcriptomic analysis of female and male adults revealed sex-biased gene expression, indicating putative genetic elements of sex determination and differentiation in this species.

**Abstract:**

*Chilo sacchariphagus* Bojer is an important sugarcane pest globally. Along with genetic modification strategies, the sterile insect technique (SIT) has gained more attention as an environment-friendly method for pest control. The identification of key genes associated with sex determination and differentiation will provide important basic information for this control strategy. As such, the transcriptome sequencing of female and male adults was conducted in order to understand the sex-biased gene expression and molecular basis of sex determination and differentiation in this species. A total of 60,429 unigenes were obtained; among them, 34,847 genes were annotated. Furthermore, 11,121 deferentially expressed genes (DEGs) were identified, of which 8986 were male-biased and 2135 were female-biased genes. The male-biased genes were enriched for carbon metabolism, peptidase activity and transmembrane transport, while the female-biased genes were enriched for the cell cycle, DNA replication, and the MAPK signaling pathway. In addition, 102 genes related to sex-determination and differentiation were identified, including the protein *toll*, *ejaculatory bulb-specific protein*, *fruitless*, *transformer-2*, *sex-lethal*, *beta-Catenin*, *sox*, *gata4*, *beta-tubulin*, *cytosol aminopeptidase*, *seminal fluid*, and *wnt4*. Furthermore, transcription factors such as *myb*, *bhlh* and *homeobox* were also found to be potentially related to sex determination and differentiation in this species. Our data provide new insights into the genetic elements associated with sex determination and differentiation in *Chilo sacchariphagus*, and identified potential candidate genes to develop pest-control strategies.

## 1. Introduction

The stem borer *C. sacchariphagus*, which lays eggs on the surface of sugarcane leaf, causes huge economic damage to sugarcane crops [1]. Currently, sugarcane borer is mainly controlled by chemicals with undesirable environmental impacts; thus, alternative environment-friendly borer control measures are being sought. The sterile insect technique (SIT) is a biological method for the control of insect pests [2,3] such as *Bactrocera tau* [4,5]. *Bombyx mori* is an important model species of Lepidoptera insects, and this transgene-based female-specific lethal system has potential for controlling lepidopteran pests such as *C. sacchariphagus* [6]. Moreover, the SIT is increasingly used to control lepidopteran pests [7]. The key to the SIT is the production of large numbers of sterile males. This is currently achieved by ionizing irradiation for classical SIT [7], or through the use of conditional lethality systems resulting in the death of male and female progeny from field-released males [8]. Additional genetic manipulations resulting in male sterility have been proposed, including the targeting of sperm-specific genes for male fertility and sex-determination genes resulting in chromosomal females developing as sterile phenotypic males [8,9].

Genes expressed in both sexes but showing relatively higher expression in one sex have been characterized as sex-biased genes [10]. These genes—which are assumed to contribute to sex differences including behavioral, physiological and morphological dimorphisms [11]—have been studied in several insects. For example, in *Drosophila melanogaster*, most differentially expressed genes were male-biased genes [12], whereas most of the sex-biased genes are female-biased in *Tribolium castaneum* and *Anopheles gambiae* [13]. In past decades, studies on sugarcane borer *C. sacchariphagus* were mainly focused on plant quarantine, integrated pest management [14,15,16], chemosensory-related genes [17], and biological characteristics and management practices [18]. However, little is known about the sex determination and differentiation mechanisms in this pest. The genes involved in sex determination and differentiation can be used for SIT [4,8,19]. Dissecting the regulatory mechanism and identifying key genes regulating sex determination and differentiation will help develop novel pest management strategies such as the SIT for controlling this insect pest.

The functions of several genes related to determination and differentiation have been elucidated in some insects. In *Drosophila melanogaster*, the sex determination cascade is known to be controlled by the *sex-lethal* gene (*Sxl*) [20]. In addition to female sex determination, *Sxl* might be related to germline development in *Bombyx mori* [21], while it is involved in oogenesis in Drosophila [21,22]. The *Sxl* is responsible for the sex-specific splicing of primary transcripts of the sex-determination master switch gene, *transformer* (*tra*) [21]. The production of female *dsx* (*doublesex*) requires both *tra* and *transformer2* (*tra2*) to form a complex and bind to the *dsx* 13-nucleotide repeated sequences (*dsxre*) [20,23,24,25]. The sex-specific splicing of *doublesex* (*dsx*) by *transformer* (*tra*) makes up a conserved gene axis [26]. Moreover, the *tra-tra2* complex is also associated with the splicing of *fruitless* (*fru*), which functions in male sexual development and in male courtship behavior in Drosophila [20,27,28]. Importantly, the primary sex determination signals showed divergent evolution in other insect species, including *Musca domestica*, *Ceratitis capitata*, *Aedes aegypti,* and *Anopheles gambiae*. For example, their *m-factors* play a key role in sex differentiation, and in mosquito species, *nix* and *yob* regulate sex differentiation by determining the male-specific splicing of *dsx* [29]. In honeybee, the *csd* (*complementary sex determiner*) gene is the initial signal for sex determination [30]. Meanwhile, *masc* and *piwi*-interacting RNA (piRNA) regulate the masculinization of embryos in silkworm [31]. Similarly, several factors—such as *wnts*, *soxs*, *gata4*, *ca* (*cytosol aminopeptidase*), *sfp* (*seminal fluid protein*) [32,33,34] and *beta-tubulin isoform* (*beta2-tubulin* in dipterans and *beta4-tubulin* in *Bombyx mori*) [9,35,36,37]—were found to modulate sexual organ development in fruit files, *A. aegypti* and other species. In brief, a large number of studies on sex determination or differentiation-related genes in mammals and Diptera insects have been reported, whereas only a few reports on Lepidoptera insects, including the model species silkworm *Bombyx mori* exist.

Transcriptome sequencing provides detailed information on all RNA molecules, such as mRNA, tRNA rRNA and noncoding RNAs [38]. It has been used for the screening of genes associated with various traits in sugarcane, such as sucrose accumulation [39,40], smut resistance [41,42] and the response to low nitrogen [43]. Transcriptome analysis was also used for the identification of sex-determining genes in many insects, such as *Plutella xylostella* [44], Phlebotominae sand flies [45], whitefly [46], *Sogatella furcifera* [47] and *Zeugodacus tau* (Walker) [48].

Sex determination and differentiation-related genes have been reported in some insects but not in sugarcane borer *C. sacchariphagus*. Hence, in this study, we analyzed the transcriptome of male and female adult sugarcane borers, and using the transcriptome dataset we explored the metabolic pathways and identified candidate genes associated with sex determination and differentiation. This work thereby presents new insights into the sex determination and differentiation in sugarcane stem borer, and also offers reference targets for the use of SIT to control this insect pest.

## 2. Materials and Methods

### 2.1. Sample Collection and Preparation

The eggs of *C. sacchariphagus*, collected from a sugarcane crop in Guangxi, China, were reared at 27 ± 1 °C with 75 ± 5% relative humidity in a 14:10 h light:dark cycle at the Sugarcane Research Institute (SRI), Guangxi Academy of Agricultural Sciences (GXAAS), Nanning, Guangxi, China. The larvae were reared on an artificial diet under the same conditions. From our experience, we noticed that the female and male adults mate at night, and after eclosion, they lay eggs mostly in the first and second night, and fewer later on. We suspected that the obvious reproductive differentiation of the sexes will have occurred in 2-day-old adults. Therefore, after successive generations, the 2-day-old females and adults were randomly selected as the female and male group samples, respectively. Each group sample included 3 individuals, and three biological replicates were sampled. All of the samples were immediately frozen in liquid nitrogen and stored at −80 °C prior to the RNA extraction.

### 2.2. RNA Isolation, cDNA Library Preparation and Sequencing

For each sample, the total RNA was isolated using a Trizol reagent according to the manufacturer’s instructions (Invitrogen, Carlsbad, CA, USA). The quantity and quality of RNA were measured by agarose gel electrophoresis and on a Bioanalyzer 2100 system (Agilent Technologies, Santa Clara, CA, USA). The concentration was checked using a Qubit^®^ RNA Assay Kit in Qubit^®^2.0 Flurometer (Life Technologies, Carlsbad, CA, USA). RNA with high purity, concentration and integrity was chosen for the cDNA library construction and further sequencing. mRNA was purified from the total RNA using magnetic beads with oligo (dT), and was then broken into small fragments. These RNA fragments were used for cDNA synthesis using a FastQuant RT Kit (Tiangen, Beijing, China). cDNA libraries were generated, and the cDNA libraries were sequenced using a BGI MGISEQ-2000 (BGI, Shenzhen, China).

### 2.3. De Novo Transcriptome Assembly and Annotation

The raw reads with adapters, primers, low-quality sequences, and ambiguous “N” nucleotides were processed to obtain the clean reads using a custom Perl script. The clean reads were assembled using Trinity v2.4.0 (min_kmer_cov:3) software [49]. Corset version 4.6 software was used to remove redundant transcripts [50]. BUSCO analysis was performed to determine the quality of the assembled transcripts [51]. Functional annotations were performed using BLASTx v2.2.28+ (e-value = 1 × 10^−5^) against public databases—including the NCBI non-reduntant protein database (Nr), Swiss-Prot database, Clusters of orthologous groups for eukaryotic complete genomes database (KOG), Kyoto encyclopedia of genes and genomes (KEGG) pathway database, and Gene Ontology (GO)—with a cutoff value of E < 10^−5^. The GO and KEGG enrichment analysis was performed using software clusterProfiler 4.0 (p AdjustMethod = “BH”) [52].

### 2.4. Identification of Deferentially Expressed Genes (DEGs)

For the discovery of DEGs between differently sexed adults, edgeR 3.0.8 (FDR < 0.05 & |log2(foldchange)| > 1) [53] was applied. The reads were mapped to the transcriptome unigenes to calculate FPKM (Fragments Per Kilobase of transcript per Million) using RSEMv1.2.15 software (mismatch 0) [54] to analyze the gene expression levels, and the DEGs were screened with a fold-change ≥2 and a false discovery rate (FDR) < 0.05.

### 2.5. Protein–Protein Interaction (PPI) Network

Protein–Protein Interaction (PPI) Network Analysis was conducted using STRING (https://string-db.org/, accessed on 17 March 2022) (e-value = 1 × 10^−10^) [55]. The DEGs were mapped onto the PPI network, and an interaction score of >0.4 was set as the threshold value. A PPI network was visualized and constructed using Cytoscape v3.6.0 [56] software. The nodes with the greatest numbers of interactions with neighboring nodes were considered to be hub nodes.

### 2.6. Validation of the Gene Expression Level by qRT-PCR

The expressions of 5 genes related to sex determination and differentiation were selected randomly, and their expressions were quantified by qRT-PCR, as described previously [40]. The reactions were performed as follows: 95 °C for 3 min, followed by 45 cycles of denaturation at 95 °C for 10 s, then annealing at 57 °C for 10 s and extension at 72℃ for 20 s. The *elongation factor 1-alpha* gene (Unigene19366_All) obtained from this transcriptome data was used as a reference. The primers used for amplification are listed in Table S1. All of the reactions were performed with 3 replicates. The threshold cycle (CT) was determined and the 2^−ΔΔCt^ equation was used to calculate the gene expression levels. The data were subjected to analysis of variance using SPSS18.0 (Analytical software, IBM, Armonk, NY, USA).

## 3. Results

### 3.1. Transcriptome Analysis of the Male and Female Chilo sacchariphagus Bojer Adults

The transcriptome sequencing generated a total of 260.53 Mb raw reads (https://ngdc.cncb.ac.cn/gsa/browse/CRA006380, accessed on 17 March 2022). After filtering, 38.50 Gb bases were retained (Table 1), from which f 60,429 unigenes were obtained (https://ngdc.cncb.ac.cn/omix/release/OMIX001036, accessed on 17 March 2022). The average length, N50, and GC content of these unigenes were 1618 bp, 3275 bp, and 38.01%, respectively. The assembled transcripts showed good BUSCO completeness, as shown in Table 2, with a complete BUSCOs ratio up to 88.7%. The functional annotation of the unigenes was performed using seven databases: NCBI non-redundant protein sequences [NR], NCBI non-redundant nucleotide sequences [NT], clusters of orthologous groups of proteins [KOG/COG], Swiss-Prot, the Kyoto Encyclopedia of Genes and Genomes (KEGG; www.genome.jp/kegg, accessed on 23 May 2022), Gene Ontology [GO], and InterPro databases. Finally, a total of 34,847 unigenes were annotated, which account for 57.67% of the total unigenes assembled (https://ngdc.cncb.ac.cn/omix/release/OMIX001092, accessed on 21 April 2022) (Table 3 and Table S2).

### 3.2. Identification of Sex-Biased Genes

**Figure 1:**
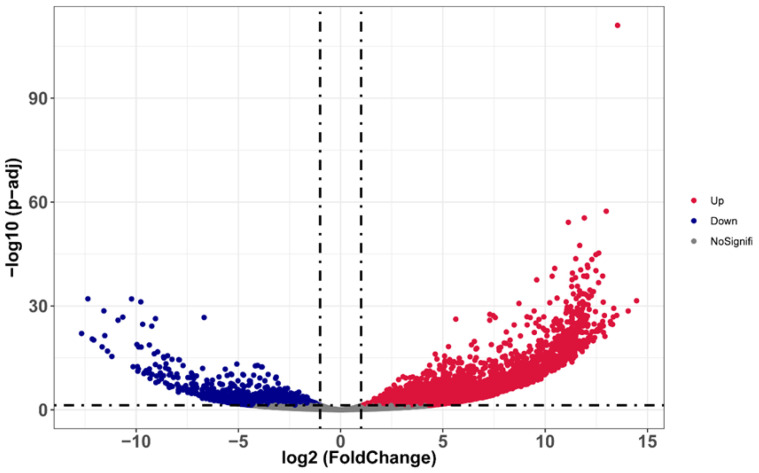
Total DEGs in the dataset. Red indicates up-regulated genes in males (male-biased genes), and blue indicates up-regulated genes in females (female-biased genes).

**Figure 2:**
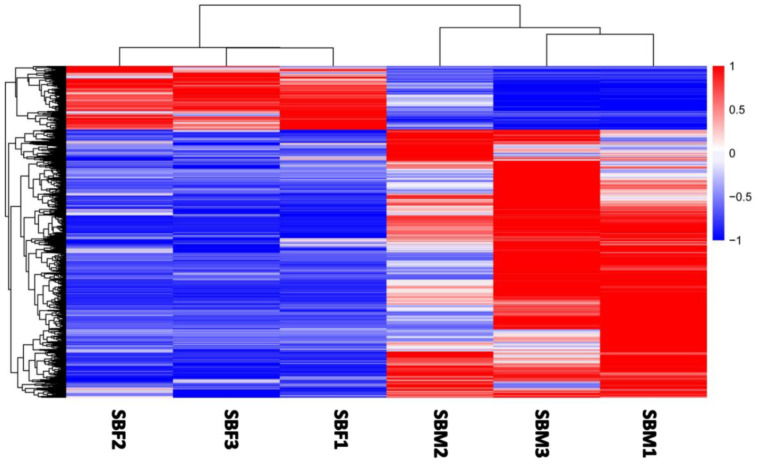
The expression trend of DEGs across all of the samples. The red color represents higher expression, and the blue color indicates lower expression.

Using the FPKM-mapped reads method, and with our assembled transcriptome as a reference, genes expressed in males and females were identified. DEGs with a >2 fold change and FDR < 0.05 were defined as significant sex-biased (SB) genes. Of the 11,121 DEGs identified, 8986 DEGs exhibited relatively higher expression levels in males than females, and they were considered to be male-biased genes (Table S3), while 2135 DEGs showed higher expressions in females, and they formed the female-biased genes (Figure 1 and Figure 2; Table S4), based on previous criteria [10].

**Figure 3:**
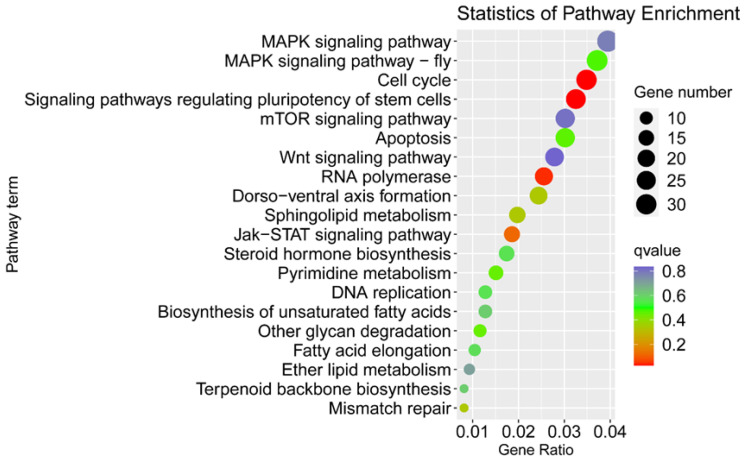
KEGG enrichment of the female-biased genes. The size of the dots corresponds to the number of DEPs in each pathway. The color displays the significance of enrichment.

In order to further elucidate the pathways in which the sex-biased genes participate, we performed KEGG analysis on the 8986 male-biased genes and the 2135 female-biased genes. Many female-biased genes were attributed to pathways related to the cell cycle, wnt signaling pathway, MAPK signaling pathway, dorso-ventral axis formation, DNA replication and RNA polymerase, etc. (Figure 3). Furthermore, these genes involved in the above-mentioned pathways have been reported to have direct or indirect effects on insect sex determination and differentiation.

**Figure 4:**
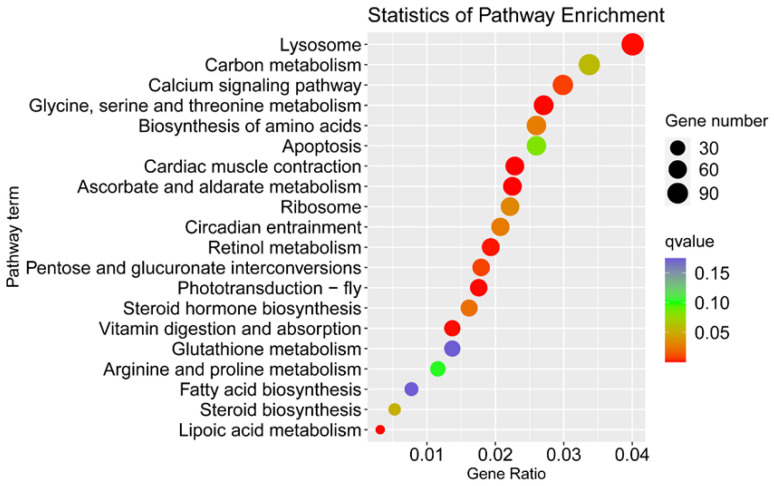
KEGG enrichment of the male-biased genes. The size of the dots corresponds to the number of DEPs in each pathway. The color displays the significance of the enrichment.

A large number of male-biased genes can be attributed to pathways associated with glycine, serine and threonine metabolism, vitamin digestion and absorption, pentose and glucuronate interconversions, the biosynthesis of amino acids, carbon metabolism, arginine and proline metabolism, and glutathione metabolism (Figure 4). These metabolic pathways may provide energy for the development and reproductive behaviour of males.

**Figure 5:**
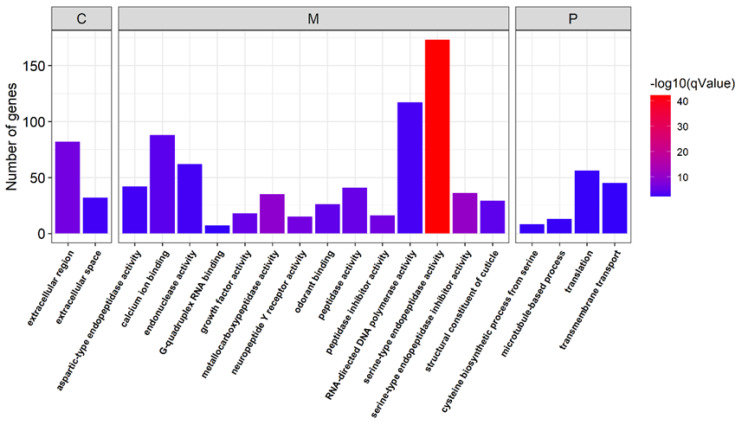
Gene ontology classification of the male-biased genes. C: cell component; M: molecular function; P: biological process.

We also conducted GO enrichment analysis to evaluate the metabolic processes that the genes are involved in. The male-biased genes were enriched in serine-type endopeptidase activity, serine-type endopeptidase inhibitor activity, neuropeptide Y receptor activity, peptidase activity, odorant binding and the transmembrane transport-related metabolic process (Figure 5).

**Figure 6:**
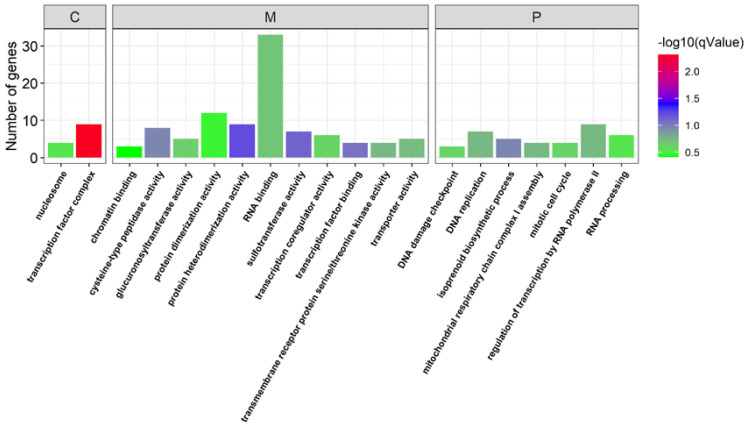
Gene Ontology classification of female-biased genes. C: cell component; M: molecular function; P: biological process.

However, female-biased genes were enriched in the metabolic processes related to transcription factor binding, the regulation of transcription by RNA polymerase II, DNA replication, transmembrane receptor protein serine/threonine kinase activity, and the mitotic cell cycle (Figure 6).

**Figure 7:**
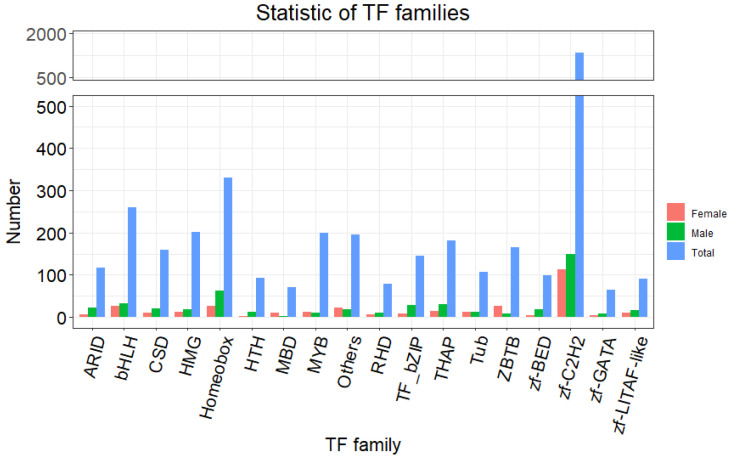
Analysis of the total and deferentially expressed transcription factors. The blue column indicates the TFs identified in this work, the red column indicates TFs expressed higher in female and the green column indicates TFs expressed higher in male.

Moreover, we found that a number of transcription factors (TFs)—such as *c2h2*, *homeobox*, *bhlh* and *thap*—were differentially associated with male and female borers, implying their role in sex determination and differentiation (Figure 7; Table S5). Interestingly, more TFs were male-biased than female-biased.

### 3.3. Identification of the Genes Related to Sex Determination and Differentiation

**Figure 8:**
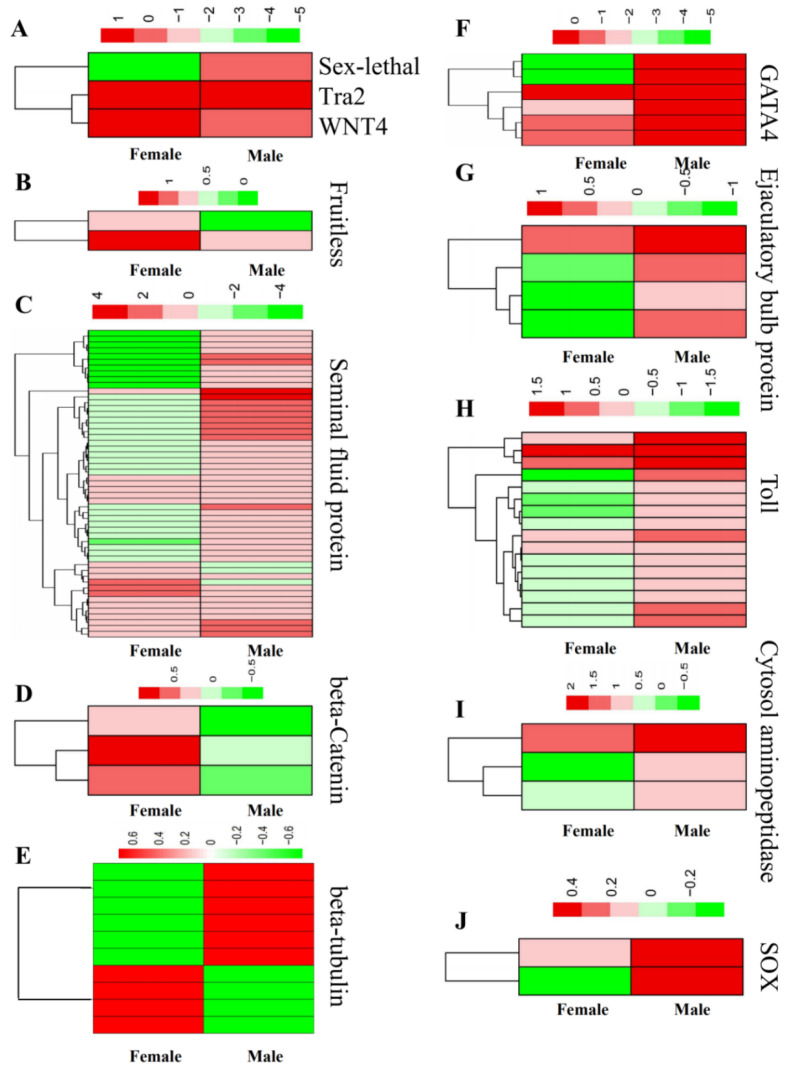
Expression of sex-determining genes in male and female borers. The different horizontal bars represent different gene IDs with the same annotation. The red and green colours indicate increased and decreased gene expression levels, respectively, following the quantitative scale (+ve and -ve numbers) shown above each heatmap. In each heatmap, the left and right columns represent the gene expressions in female and male adults, respectively. The expression patterns of *sex-lethal*, *tra2* and *wnt4* (**A**), fruitless (**B**), *seminal fluid proteins* (**C**), *beta-catenin* (**D**), *beta*-*tubulin* (**E**), *gata4* (**F**), *ejaculatory bulb-specific protein* (**G**), *toll* (**H**), *cytosol aminopeptidase* (**I**) and *sox* (**J**) in the female and male adults.

The core genes related to sex determination and differentiation are conserved among the arthropods, and the functions of some of these genes have been elucidated [23,24,25,26,47]. Therefore, with the annotation of the sex-related genes identified in our study, we searched for the sex determination and differentiation genes in sugarcane borers. Overall, we identified 110 sex determination and differentiation genes (Table S6), including protein *toll*, *ejaculatory bulb-specific protein 3*, *fruitless*, *transformer-2* (*tra2*), *sex-lethal*, *beta-catenin*, *sox*, *gata4*, *beta-tubulin*, *cytosol aminopeptidase*, *seminal fluid protein* and *wnt4*. Among them, *beta-catenin*, *wnt4* and *tra2* showed higher expression in female borers, while *sex-lethal*, *gata4*, *ejaculatory bulb-specific protein 3*, *toll* and *cytosol aminopeptidase* had higher expression in male borers. Besides this, most of the *seminal fluid protein* and *beta-tubulin* genes were expressed more abundantly in male rather than female borers (Figure 8).

### 3.4. Construction of the Gene Network Involved in Sex Determination and Differentiation

**Figure 9:**
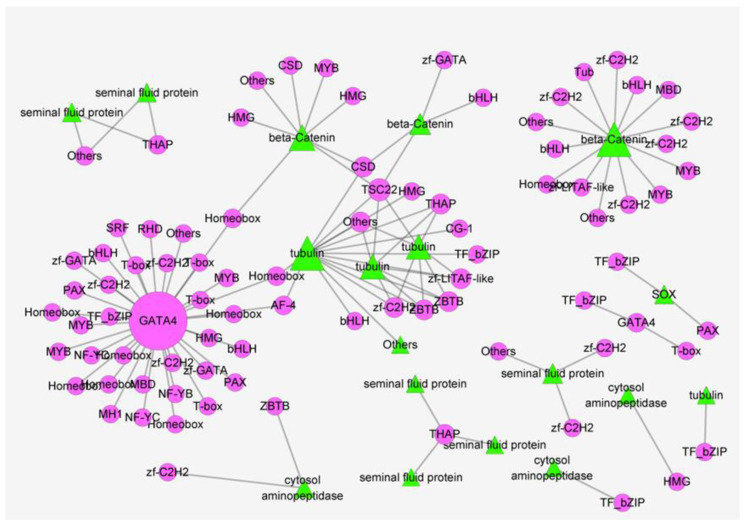
PPI network of sex determination and differentiation-related genes in *Chilo sacchariphagus*.

In order to explore the interaction network among sex determination and differentiation-related genes in sugarcane borer, we performed a protein–protein interaction (PPI) network analysis using the STRING database [55] and Cytoscape [56] on the 110 sex determination and differentiation genes and the TFs identified in this work. The PPI network contained 296 nodes and 104 edges. According to the PPI network, six genes (degree ≥7) were selected as hub genes, including *gata4* (Unigene10060_All), *beta-tubulin* (Unigene7114_All/CL2807.Contig1_All/Unigene14411_All) and *beta-catenin* (CL2862.Contig1_All/CL4096.Contig1_All). Among them, *gata4* had the highest degrees (with the most connected genes), suggesting that it may play a crucial role in sex determination and differentiation in sugarcane borer. In addition, some TFs—including *myb*, *bhlh* and *homeobox*—were also present in the PPI network, indicating their involvement in sex determination and differentiation in *C. sacchariphagus* (Figure 9).

### 3.5. Verification of DEGs Using qRT-PCR

**Figure 10:**
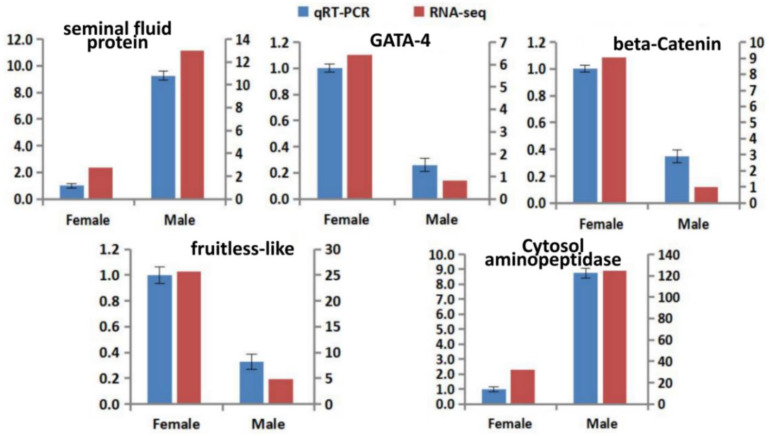
Expression levels of the genes given by high-throughput sequencing and qRT-PCR. The Y axis (left) indicates the relative expression by qRT-PCR, and the Y axis (right) indicates the FPKM caculated by RNA-seq.

In order to validate the reliability of the transcriptome sequencing data, the expressions of five genes (CL1718.Contig1_All/*fruitless-like*, CL4096.Contig1_All/*beta-catenin*, Unigene6400_All/*cytosol aminopeptidase*, Unigene11772_All/*seminal fluid protein* and CL6380.Contig6_All/*gata4*) were randomly selected from the 102 sex-determining and sex-differential genes identified in this work, and were analysed by qRT-PCR, with the elongation factor 1-alpha gene (Unigene19366_All) as reference. The dissolution curve of all of the primers had a single peak without any miscellaneous peak. The amplification efficiency of the reference gene was 98%. The relative mRNA expression of the unigenes assessed by qRT–PCR (Figure 10) was very similar to the levels shown in the RNA-seq analysis, suggesting the reproducibility and accuracy of the RNA-seq results.

## 4. Discussion

Sugarcane borer, *C. sacchariphagus*, is a major agricultural pest causing severe economic loss to the sugarcane industry [1]. The SIT is an environment-friendly and efficient technique to control insect pests [2,3]; it has been successfully used to control lepidopteran pests, and provides insights into field performance [7]. The identification of sex determination and differentiation genes will facilitate the development of the SIT for borer control in sugarcane. Transcriptomic analysis has become an effective method for the analyzing differential gene expression, discovering new genes and obtaining transcriptional information related to vital traits [38,57]. In this study, we analyzed the female and male adults of *C. sacchariphagus* transcriptome. A total of 11,121 DEGs were identified, of which, 8936 DEGs were male-biased genes and 2135 DEGs were female-biased, indicating that more genes were upregulated in male than in female sugarcane borers. This is a common phenomenon in insects [58,59].

Gene Ontology (GO) analysis showed that the identified sex-biased genes were highly enriched in the processes associated with reproductive functions. For example, male-biased genes were enriched in the process of transmembrane transport, which may be mainly associated with the motile nature of sperm [12], and were also enriched in serine-type endopeptidase activities. These enrichments seen in borer are similar to those seen in the insects *Plutella xylostella* and *Aedes aegypti* [9,44]. Serine-type endopeptidase activity is expected to be associated with the development of sex-specific organs [9]. Microtubule-based process and calcium ion binding proteins were also found to be enriched in male adult sugarcane borer, which is consistent with the findings in *Aedes aegypti* [60]. This result can be explained by the function of microtubule-based processes, which are associated with spermatogenesis and sperm composition [9], and calcium-dependent regulation, which is necessary for normal sperm motility [60].

Furthermore, similarly to previous studies in Drosophila, mosquitoes and Daphnia [61,62,63], female-biased genes in sugarcane borer were enriched in the process of DNA replication and the component of nucleosome. DNA replication was required for egg production [12,64,65], and may also be related to maternally deposited gene products [12]. Nucleosomes have an important role in the development of sex-specific organs [9].

KEGG enrichment results provided molecular insights into the sexually dimorphic traits of *C. sacchariphagus*. In this study, female-biased genes are mainly associated with the cell cycle, DNA duplication and carbohydrate biosynthesis, which may facilitate the reproductive ability of females. However, male-biased genes were enriched in pathways including carbon, lipoic acid, glycine, serine, threonine, ascorbate, aldarate, arginine and proline metabolism. They may provide energy for the development of males.

Based on the sex determination and differentiation-related genes reported in other insects [66,67,68], we searched our DEGs and identified 110 sex determination and differentiation-related genes from *C. sacchariphagus*, including *protein toll*, *ejaculatory bulb-specific protein 3*, *fruitless*, *transformer-2*, *sex-lethal* (*Sxl*), *beta-catenin*, *sox9*, *gata4*, *beta-tubulin*, *cytosol aminopeptidase*, *seminal fluid protein* and *wnt4*. Among them, some were related to female determination, such as *Sxl*, *wnt4* and *bata-catenin*, and some were related to male determination, including *sox* and *gata4*. Some sex-biased genes were associated with sex organ components and development, including cytosol aminopeptidase protein and seminal fluid protein. In *Drosophila melanogaster*, sex determination is controlled by five main genes—*sex-lethal* (*Sxl*), *transformer* (*tra*), *transformer-2* (*tra-2*), *doublesex* (*dsx*) and *fruitless* (*fru*)—hierarchically organized in a regulative cascade: *Sxl* − > *tra* + *tra-2* − > *dsx*, *fru* [20,69,70]. Furthermore, the functions of these genes in sex determination have also been identified in other insect orders, such as Diptera, Coleoptera, Lepidoptera and Hymenoptera [30,71,72]. In the sex determination signaling pathway in *Drosophila melanogaster*, *Sxl* regulates its own pre-mRNA splicing, and the splicing of tra is controlled by *Sxl* [66]. In silkworm, *Bombyx mori*, a different signal casade related to sex determination and differentiation consisting of Feminizer piRNA, Masculinizer, Argonaute RISC Catalytic Component 3, and female-specific *dsx* forms the primary components [73]. In our work, *tra2* showed higher expression in females, but *Sxl* was expressed more abundantly in males than in females. As such, further study is proposed to verify whether the activation of the *tra2* only occurs in female *C sacchariphagus* and *Sxl* only occurs in male borers. DEGs associated with sex signal casades in silkworm were not found in our *C sacchariphagus* transcriptomic analysis, suggesting potential functional divergence in sex signalling in Lepidoptera insects.

When we focused on sex differentiation, we found genes involved in sexual organ components and development. *gata4*, *beta-catenin*, *wnt4*, *sox9*, *beta-tubulin*, *ca* and *sfp* were found to be differentially expressed in female and male adults. *gata4* is a key regulator of male sex differentiation and determination, including testis differentiation, in mammals [74]. In mice, *gata4* is down-regulated in female embryonic gonads [67]. *gata4* suppresses ovary-specific genes and *blastoderm-specific protein 25D* [75]. However, in our work, *gata4* showed higher expression in female adult borers than in males, implying that the gene *gata4* has a more complex function in sex determination in this species.

*beta-catenin* was present in the gonads of both sexes, and it was necessary for ovarian differentiation in mice [68]. The expression of *Wnt4* becomes ovary-specific at sex determination in mammals [76]. The *bmix* female mutants were sterile and had irregular external genitalia, and their *Bmwnt1* activity was significantly downregulated [77]. We showed that *wnt-2* contributes to the survival of a male-specific population of somatic gonadal precursor cells (*SGPs*) in *Drosophila melanogaster* [78]. *wnt*/*β-catenin* signaling regulates *Helicoverpa armigera* pupal development [79]. In our work, *beta-catenin* and *wnt4* showed higher expression in female adult borer, suggesting that they may be necessary to female germ cell survival and ovary development [77].

The *sox9* gene is essential for testis development, and is expressed in a male-specific manner which is related to primary sex determination in humans [80]. We have found that the Drosophila embryonic gonad is already sexually dimorphic at the time of initial gonad formation. Male-specific somatic gonadal precursors (*msSGPs*) contribute only to testis development, and the expression of one of them—a Drosophila homolog of *sox9* (*sox100B*)—is essential for testis formation in humans [80]. *tub* (*beta-tubulin*), *ca* (*cytosol aminopeptidase*) and *sfp* (*seminal fluid protein*) are up-regulated in males, which has an important functional effect on male fertility [32,33,34]. The testis-specific *β2-tubulin* gene in *D. melanogaster* has been determined to function in spermatogenesis [35]. Mutant *β2-tubulin* disrupts sperm development and causes male sterility [36]. The cytosol aminopeptidase protein was found to be an *A. aegypti* sperm composition protein [8]. In insects, *SFP* is produced in the male accessory glands, which significantly increases male fitness [37]. In our work, *sox9*, *tub*, *CA* and *sfp* were mostly abundant in male rather than female borers, implying that they may play a role in male fertility.

Expression analysis related to sex-determining genes in *C. sacchariphagus* was also analysed in Liu et al.’s studies [81]. There are many important differences between our results and Liu et al.’s findings. Firstly, in the KEGG and GO analysis of Liu et al. [81], “signal transduction mechanisms”, “posttranslational modification, protein turnover, chaperones”, and “environmental information processing” appeared as the main enriched pathways. However, in our work, “cell cycle”, “wnt signaling pathway”, “MAPK signaling pathway”, “dorso-ventral axis formation”, “DNA replication” and “RNA polymerase”, “lycine, serine and threonine metabolism”, “vitamin digestion and absorption”, and “pentose and glucuronate interconversions” emerged as the main pathways in which the sex-bias genes mainly participate. This is a major and significant difference in the experimental outcomes between these two studies. Secondly, the DEGs in our results are greatly different from those reported by Liu et al. They focused on the genes *tra2*, *dsxF*, *dsxM*, *emc*, *gro X4*, *gro X9*, *ix*, *fru*, *da X3*, *fl(2)d*, *vir*, *Sxl*, *runt*, *fem*, *csd*, and *rbp1*. However, the key DEGs we found were *ejaculatory bulb-specific protein 3*, *fruitless*, *transformer-2*, *sex-lethal*, *beta-catenin*, *sox*, *gata4*, *beta-tubulin*, *cytosol aminopeptidase*, *seminal fluid protein* and *wnt4*, and those related to the protein *toll*. Thirdly, we additionally identified TFs relevant to sex determination and differentiation, and used protein–protein interaction analysis to reveal the regulatory networks of transcription factors and sex determination-related genes, providing new insights into sex-related gene regulation. Thus, our research presents important new knowledge in this area, complementary to the previous study, and provides new sights into the molecular elements of sex determination and development of this major sugarcane pest.

**Figure 11:**
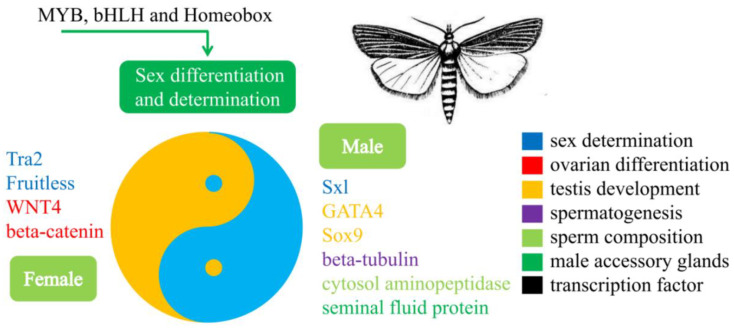
Genes related to sex determination and differentiation in *Chilo sacchariphagus*. The genes listed on the left of the circle showed higher expression in females, and the ones on the right side showed higher expression in males.

Taken together, the sex determination and differentiation-related genes identified in this study are candidate targets for the development of SIT for controlling *C. sacchariphagu*. In this respect, *gata4*, *beta-tubulin*, *beta-Catenin*, *myb* and *bhlh* are particularly valuable because they form the hub genes in the sex-related regulatory network. Based on these findings, a putative model is proposed (Figure 11). This includes *fruitless*, *transformer-2* and *sex-lethal* as sex-determining factors; *wnt4* and *beta-catenin* for ovarian differentiation; and *gata4* and *sox9* for testis development, *beta-tubulin* for spermatogenesis, and *cytol aminopeptidase* and *seminal fluid protein* for male accessory components. Here, it is also worth noting that the *myb*, *bbhlh* and *homeobox* TFs could be controlling sex determination and differentiation by regulating the activity of the above-mentioned genes. The genetic manipulation-based SIT has been used successfully in Lepidoptera moth insects, such as the diamondback moth [7], pink bollworm [82] and silkworm [6], suggesting the possibility of SIT for sugarcane borer control. Moreover, we used RNAi to silence genes in sugarcane borer, which further strengthen the application of SIT in sugarcane borer for male feminization, female masculinization and impaired reproduction ability. Clearly, a gene shows functional divergence in different species [29,30,31]. As such, more experiments are needed in order to determine candidate gene functions in sugarcane borer unequivocally.

## 5. Conclusions

Sugarcane borer, *C. sacchariphagus* Bojer is an important sugarcane pest. In order to understand the molecular basis of sex determination and differentiation, and to provide targets for the development of the sterile insect technique, we performed transcriptome sequencing of six cDNA libraries of female and male adults of *C. sacchariphagus*. We assembled 60,429 unigenes, of which 11,121 were DEGs between males and females, with male-biased genes being four-fold more numerous than female-biased genes. Sex-biased genes are very likely to be involved in reproductive functions and the sexually dimorphic traits of *C. sacchariphagus*. We also identified 102 sex determination and differentiation-related genes, including protein *toll*, *ejaculatory bulb-specific protein 3*, *fruitless*, *transformer-2*, *sex-lethal*, *beta-catenin*, *sox*, *gata4*, *beta-tubulin*, *cytosol aminopeptidase*, *seminal fluid protein* and *wnt4*. Moreover, TFs *myb*, *bhlh* and *homeobox* may also be potentially related to sex determination and differentiation in sugarcane borer (Figure 11). This work, for the first time, reported potential molecular regulators and regulatory mechanisms of sex determination and differentiation in sugarcane borer. It also presents target candidate genes for the further functional dissection of sex determination and differentiation phenomena, and to develop control strategies for this important sugarcane pest.

## Figures and Tables

**Table 1:** Summary of the *C. sacchariphagus* transcriptome.

Sample	Raw Read Number	Raw Read Base Number	Clean Reads Base Number	Clean Read Q20 (%)	Clean Read Q30 (%)	Clean Read Ratio (%)
SBF1	43.82 Mb	6.57 Gb	6.48 Gb	96.1	90.65	98.58
SBF2	43.82 Mb	6.57 Gb	6.51 Gb	95.98	90.41	99.01
SBF3	41.43 Mb	6.21 Gb	6.1 Gb	96.05	90.58	98.21
SBM1	43.82 Mb	6.58 Gb	6.51 Gb	96.16	90.78	98.98
SBM2	43.82 Mb	6.57 Gb	6.49 Gb	95.94	90.27	98.78
SBM3	43.82 Mb	6.57 Gb	6.41 Gb	96.29	91.08	97.57

**Table 2:** BUSCO analysis of the assembled transcripts.

Category	Number	Ratio
Complete BUSCOs (C)	1471	88.70%
Complete and single-copy BUSCOs (S)	812	49%
Complete and duplicated BUSCOs (D)	659	39.70%
Fragmented BUSCOs (F)	22	1.30%
Missing BUSCOs (M)	165	10.00%
Total BUSCO groups searched	1658	100%

**Table 3:** Summary of the annotations of the assembled *C. sacchariphagus* unigenes.

Database	Number	Percentage
NR	32,735	54.17%
NT	15,948	26.39%
Swiss-prot	22,226	36.78%
KEGG	24,875	41.16%
GOG	21,590	35.73%
Pfam	23,379	38.69%
GO	15,013	24.84%
Total unigenes	60,429	100%
Annotated unigenes	34,847	57.67%

## Data Availability

The datasets generated and/or analysed during the current study are available from the Genome Sequence Archive in the National Genomics Data Center, China National Center for Bioinformation/Beijing Institute of Genomics, Chinese Academy of Sciences (GSA ID: CRA006380; https://bigd.big.ac.cn/gsa/browse/CRA006380, accessed on 17 March 2022).

## Supplementary Materials

The following supporting information can be downloaded at https://www.mdpi.com/article/10.3390/insects13060500/s1. Table S1: Primers used for qRT-PCR; Table S2: List of the total annotated unigenes; Table S3: List of male-biased genes; Table S4: List of female-biased genes; Table S5: List of transcription factors which showed differential expression between males and females; Table S6: List of sex-determining genes identified in this work.
